# Spatiotemporal Trends in Deaths From External Causes in Brazil: 23-Year Ecological and Population-Based Study

**DOI:** 10.2196/68002

**Published:** 2025-09-15

**Authors:** Lucas Almeida Andrade, Wandklebson Silva da Paz, Luís Ricardo Santos de Melo, Débora dos Santos Tavares, Alvaro Francisco Lopes de Sousa, Emerson Lucas Silva Camargo, Carla Aparecida Arena Ventura, Regina Claudia Silva Souza, Karina Conceição Gomes Machado de Araújo, Carlos Dornels Freire de Souza, Allan Dantas dos Santos, Fagner Alfredo Ardisson Cirino Campos, Márcio Bezerra Santos

**Affiliations:** 1 Graduate Program in Health Sciences, Universidade Federal de Sergipe, Rosa Elze, São Cristóvão, Aracaju, 49100000, Brazil, 55 16982198803; 2 Graduate Program in Tropical Medicine, Center for Medical Sciences, Universidade Federal de Pernambuco, Recife, Brazil; 3 Graduate Program in Nursing, Universidade Federal de Sergipe, São Cristovão, Brazil; 4 Department of Health Education, Federal University of Sergipe, Lagarto, Sergipe, Brazil; 5 Institute of Teaching and Research, Hospital Sírio-Libanês, São Paulo, Brazil; 6 Public Health Research Centre, Comprehensive Health Research Center (CHRC), NOVA National School of Public Health, Universidade Nova de Lisboa, Lisbon, Portugal; 7 Ribeirão Preto College of Nursing, Universidade de São Paulo, Ribeirão Preto, Brazil; 8 College of Medicine, Universidade Federal do Vale do São Francisco, Petrolina, Brazil; 9 Department of Nursing, Federal University of Sergipe, Lagarto, Sergipe, Brazil; 10 Complex of Medical and Nursing Sciences, Federal University of Alagoas, Arapiraca, Alagoas, Brazil

**Keywords:** mortality, accidents, violence, Brazilian, deaths, young adults, mortality information system, SIM, joinpoint regression, Moran's I, Poisson scanning statistics, population-based study, spatial analysis, public health, spatiotemporal analysis, historical series, self harm, assaults, temporal trends, external causes

## Abstract

**Background:**

Mortality from external causes is a major public health issue globally, with significant impacts on both lives and economies. In Brazil, external cause mortality has shown spatiotemporal variations across regions, influenced by social, economic, and demographic factors.

**Objective:**

This study aimed to examine the spatiotemporal dynamics of mortality from external causes in Brazil for 23 years (2000‐2022), identifying patterns across regions and demographic groups and assessing the major contributing causes of death.

**Methods:**

This ecological study used data from the Brazilian Mortality Information System (SIM) and used joinpoint regression to analyze temporal trends, Moran I for spatial analysis, and Poisson scanning statistics for spatiotemporal patterns. A total of 3,240,023 deaths were analyzed, with specific attention given to regional and demographic disparities.

**Results:**

The study found that mortality from external causes remained significant, with men and young adults (20‐39 years) having the highest rates of death. The main causes of death were assaults (36.61%), transport accidents (26.55%), falls (7.83%), and self-harm (7.43%). Despite an overall decrease in mortality, increases were observed in the North and Northeast regions and among the older adults. High-risk areas were predominantly located in the North, Northeast, and Central-West regions. The mortality trends varied by region, with significant differences in risk across the country.

**Conclusions:**

Although there was a general reduction in mortality from external causes in Brazil, this trend was not uniform across all regions. The North, Northeast, and Central-West regions showed the highest mortality risks, with men and young adults being the most affected demographic groups. These findings emphasize the need for targeted public health interventions that address the regional and demographic disparities in mortality from external causes.

## Introduction

Mortality from external causes represents one of the main global public health problems. This phenomenon encompasses both unintentional events, such as traffic accidents, drownings, and falls, as well as intentional events, resulting from injuries caused by different forms of violence, such as suicide, homicide, femicide, and others [1]. Notably, these causes are impactful not only because of the number of lives lost but also due to the high socioeconomic burden they impose on countries and the negative consequences for affected families and health systems [1,2].

According to data from the World Health Organization (WHO), more than 4 million people die annually from external causes worldwide (about 8% of all global deaths) [1,3]. The WHO estimates that 29% of these fatal outcomes result from traffic accidents, 16% from suicides and falls, and 11% from homicides. Furthermore, teenagers and young adults aged 15-29 years are the most affected [3].

In Brazil, external causes constitute a significant issue in national mortality rates and represent substantial challenges to the national health system, being one of the major causes of disability and premature mortality [4,5]. In 2021, deaths from violence and accidents accounted for approximately 8% of all deaths across the country, ranking as the fourth leading cause of mortality, with particular emphasis on homicides and transport accidents, which together were responsible for more than 50% of deaths related to external factors in Brazilian territory [5].

Despite the significant socioeconomic impacts of accidents and violence worldwide, certain population groups and regions are more vulnerable [6,7]. Mortality patterns from external causes can be influenced by social, economic, and demographic determinants, such as income, age structure, the level of socioeconomic inequality, and the presence and efficiency of public policies in the areas of education, health, and safety [1,8]. In this context, deaths from external causes are not evenly distributed across Brazil and may present marked spatial heterogeneity across regions of the country [7,9,10]. This is due to Brazil’s complex reality, which is manifested through distinct geographic, cultural, political, and socioeconomic characteristics, creating different mortality patterns throughout the country [11].

Importantly, in 2011, the Brazilian Ministry of Health launched the Strategic Action Plan to Combat Chronic Noncommunicable Diseases (NCDs) (2011‐2022), with emphasis on effective, integrated, and sustainable strategies for the prevention and control of noncommunicable diseases and their risk factors. Nevertheless, as the plan’s end year approached (in 2019), the Brazilian Ministry of Health began preparing a new document that reaffirmed and expanded the established proposals. The DANT Plan (Strategic Action Plan to Combat Chronic Diseases and Non-Communicable Diseases in Brazil, 2021‐2030) also included external causes of death. This collective effort is part of the health agenda for the next 10 years, in line with the 2020‐2030 Agenda on Sustainable Development Goals. The goals aim to reduce the mortality rate from traffic injuries by 50%, reduce the homicide mortality rate by one-third, and halt the growth of mortality from suicides and fall-related deaths among older adults in Brazil by 2030 [2].

In this regard, combating deaths from external causes requires a range of approaches that strengthen prevention and control strategies in Brazil. Therefore, spatial analysis techniques using geographic information systems are fundamental tools [12], as they provide a better understanding of the spatial dynamics of violent and accidental incidents, identifying high-mortality areas that require priority investment for effective interventions and public policies that consider the health realities of each region. This study aimed to analyze the spatiotemporal dynamics of mortality from external causes in Brazil and its regions for 23 years.

## Methods

### Type and Study Design

An ecological and population-based study was conducted using spatiotemporal analysis techniques. The study encompassed all deaths related to external causes in Brazil from 2000 to 2022. This time frame was established because, starting in 1999, Brazil adopted a new version of the Mortality Information System (SIM), which introduced an updated death certificate (DC). This update improved data recording, with more detailed completion of the DC [13]. When the data were collected for this study, the numbers of deaths from external causes were only available up to 2022. The 5 regions of the country and their 5570 municipalities were considered for all analyses.

### Study Area

**Figure 1.**
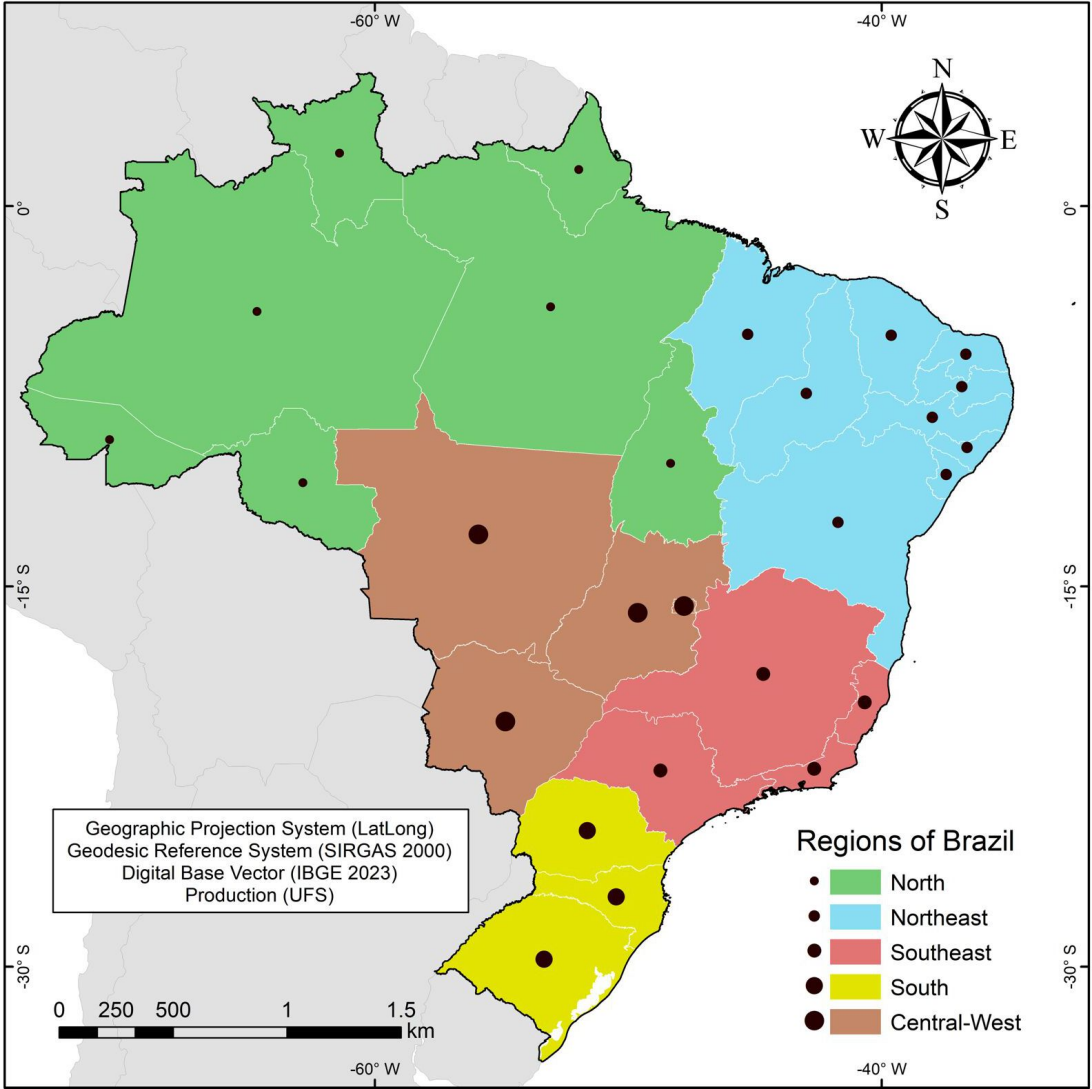
Study area: a map of Brazil divided into its 5 regions, 26 states, and 1 federal district. Coordinates based on latitude and longitude. Map elaborated by Universidade Federal de Sergipe. IBGE: Instituto Brasileiro de Geografia e Estatística; LatLong: latitude and longitude; SIRGAS: Sistema de Referência Geocêntrico para as Américas; UFS: Universidade Federal de Sergipe.

Brazil is located in South America, being the largest country on the continent, with a territorial area of 8,515,767.049 km². The Brazilian population is approximately 203 million inhabitants. The country is politically and administratively divided into 27 federative units (26 states and 1 federal district), with the capital city being Brasília. For political and operational purposes, the federative units are grouped into five regions (North, Northeast, Southeast, South, and Central-West) with distinct geographic, socioeconomic, and cultural characteristics [11] (Figure 1).

### Data Source

Data regarding deaths from external causes were collected from the Brazilian SIM. The SIM plays a fundamental role in the process of collecting, storing, and managing death records in the country, using the DC as a standard document, which is a form filled out by medical professionals for all deaths occurring in Brazil. It is important to highlight that SIM data are in the public domain and available for access on the website of the Department of Informatics of the Unified Health System. For data acquisition, codes V01 to Y98 from the *ICD-10* (*International Statistical Classification of Diseases and Related Health Problems, Tenth Revision*) were used.

In addition, population data were obtained from the Brazilian Institute of Geography and Statistics, based on information from the Brazilian population censuses conducted in 2000, 2010, and 2022, as well as intercensal population estimates for the years 2001‐2009 and 2011‐2019 [12]. The digital cartographic mesh of Brazil (divided by states and regions), based on the Geographic Projection System in shapefile format (Geodesic Reference System, SIRGAS/2000), was extracted from the Brazilian Institute of Geography and Statistics website for the preparation of spatial analysis maps.

### Variables and Measures

The variables assessed were (1) number of deaths from external causes registered in the 5570 Brazilian municipalities; (2) age-standardized external cause mortality rates (ASMRs) based on the 1966 world standard population, as defined by Doll et al [14]. This standardization allows for data comparison at national and international levels, accounting for differences in the age pyramids of diverse geographic areas and over time. ASMR rates were calculated in age-specific ranges (0‐4, 5‐9, ..., 75‐79, and ≥80 years). The result was multiplied by 100,000 inhabitants in each municipality, state, and region. Rates were also calculated according to sex and age group.

### Exploratory Data Analysis

The epidemiological variables used in the descriptive analysis were: sex (male and female), ethnicity/skin color (“White,” “Black,” “Yellow,” “Brown,” and “Indigenous”; in the Brazilian context, the terms White [*branco*], Black [*preto*], Brown [*pardo*], Yellow [*amarelo*], and Indigenous [*indígena*] are the official racial/ethnic categories established by the Brazilian Institute of Geography and Statistics and are systematically used in national censuses and health information systems, including the SIM), age group (0-9, 10-19, 20-39, 40-59, and ≥60 years), years of education (<8 and ≥8 years), and cause of death (according to the *ICD-10* code). These categorized variables were described for Brazil and its regions using absolute and relative frequencies.

### Time Trend Analysis

Temporal trends were assessed through the joinpoint linear regression model (segmented linear regression), using standardized mortality rates from external causes by region, gender, and age group. This method allowed for the verification of changes in data trends over time by adjusting the data to a time series with the smallest possible number of joinpoints (zero, which indicates a line without inflection points) and testing whether the inclusion of more joinpoints was statistically significant [15].

The statistical significance test for choosing the best model was based on the Monte Carlo permutation method, considering *P*<.05 and 95% CI. To describe and quantify the trends, the annual percentage changes (APCs) and their respective 95% CIs were calculated. The average annual percentage change (AAPC) was calculated for the total period when more than one APC was obtained for segmented periods. The AAPC value was derived from the weighted geometric mean of the APCs, with weights corresponding to the length of each segment’s time interval [16]. Trends were considered significant when APCs presented *P*<.05 and their 95% CI did not include a zero value. A positive and significant APC value indicated an increasing trend, while a negative and significant value indicated a decreasing trend. Nonsignificant trends were described as stable, regardless of APC values [16].

### Spatial Analysis and Spatiotemporal Scanning

For spatial analyses, choropleth maps of Brazil were created to represent mortality from external causes across all municipalities. Initially, standardized mortality rates were used, but to minimize the instability caused by random fluctuations in cases, the local empirical Bayesian estimator was applied. This model smoothed the standardized rates through weighted averages, resulting in a new corrected coefficient that more accurately represented the epidemiological scenario [17]. This method also reduces data fluctuations in smaller areas. One of the advantages of Bayesian rates is the attribution of greater influence to neighboring municipalities, making the results more coherent at a regional level. To facilitate data visualization, the standardized and smoothed rates were presented in thematic maps, stratified into five categories of natural breaks (Jenks): (1) 0‐45, (2) 45‐61, (3) 61‐77, (4) 77‐97, and (5) 97‐192 deaths per 100,000 inhabitants [17].

Subsequently, the Global Moran Index was calculated to investigate the existence of spatial autocorrelation in mortality from external causes and to determine if spatial patterns were present in the distribution of this variable. The Global Moran Index estimates the correlation between the values of a variable in different locations, ranging from −1 to +1. Values close to 0 indicate the absence of spatial autocorrelation, while positive values (0 to +1) indicate the presence of positive spatial autocorrelation. That is, areas with high values tend to be close to other areas with high values, and areas with low values tend to be close to other areas with low values. On the other hand, negative values (−1 to 0) indicate negative spatial autocorrelation, where areas with high values are close to areas with low values [17,18].

Once autocorrelation was identified, the occurrence of local autocorrelation was assessed by calculating the Local Moran Index (local indicators of spatial association), which determined the existence of patterns of spatial dependence. A scatter plot was also created with the following spatial quadrants: Q1 (high and high) and Q2 (low and low), indicating municipalities with similar values to their neighbors and with positive spatial association; Q3 (high and low) and Q4 (low and high), indicating municipalities with different values from their neighbors without spatial association. The results were considered statistically significant when *P*<.05 and represented in Moran maps [17,18].

Finally, spatiotemporal scanning analysis was applied to identify and evaluate spatiotemporal clusters of high risk for deaths from external causes. The identification of clusters occurred through scanning statistics (SaTScan; Kulldorff, 1997), using the retrospective space-time analysis type, through the Poisson probability distribution model, which met the following parameters: aggregation time of one year, no geographic or temporal overlap of clusters, circular clusters, maximum spatial cluster size of 50% of the population at risk, and a maximum temporal cluster size equal to 50% of the studied period [19]. Spatiotemporal clusters were detected using the log likelihood ratio test. Furthermore, the relative risks of mortality were calculated for each cluster in relation to its neighbors. Results with a *P*<.05 were considered significant, using 999 Monte Carlo simulations, and were represented in the form of maps and tables [19].

### Software

Microsoft Excel 2017 was used for data tabulation and descriptive analysis, Joinpoint Regression Program 5.0.2 (Statistical Methodology and Applications Branch, Surveillance Research Program, National Cancer Institute) was used to assess time trends, TerraView 4.2.2 (Instituto Nacional de Pesquisas Espaciais – INPE, Brazil) was used to perform spatial analysis, QGIS 3.28.7 (QGIS Development Team, Open Source Geospatial Foundation) was used to create choropleth maps, and SaTScan 9.6 (developed by Martin Kulldorff and colleagues, Harvard Medical School/Brigham and Women’s Hospital, USA) was used to perform spatiotemporal scanning.

### Ethical Considerations

This study used secondary data from the public domain, which did not contain any personal identifiers, and followed national and international ethical recommendations, including the rules of the Declaration of Helsinki and Resolution 466/2012 of the National Health Council.

## Results

A total of 3,240,023 deaths related to external causes were registered in Brazil from 2000 to 2022. The regions with the highest percentages of deaths were the Southeast (n=1289,073, 39.79%) and the Northeast (n=945,850, 29.19%), together representing 68.98% of the total deaths due to external factors in the country. When assessing the sociodemographic characteristics of these deaths, the majority was among men (n=2,668,434, 82.36%), individuals identified as non-White (n=1,795,050, 55.11%), those aged between 20 and 39 years (1,432,969, 44.23%), and individuals with fewer than 8 years of education (n=1,554,174, 47.97%). Likewise, all Brazilian regions exhibited this profile described above, except for ethnicity in the Southeast and South regions, where deaths from external causes were higher among White individuals (n=656,634, 50.94% and n=385,393, 83%, respectively; Table 1).

**Table 1.** Sociodemographic characteristics of deaths from external causes in Brazil, 2000-2022.

Variable	North, n (%)	Northeast, n (%)	Southeast, n (%)	South, n (%)	Central-West, n (%)	Brazil, n (%)
Total	267,757 (8.26)	945,850 (29.19)	1,289,073 (39.79)	464,354 (14.33)	272,989 (8.43)	3,240,023 (100)
Sex						
Male	229,908 (85.86)	805,311 (85.14)	1,036,003 (80.37)	373,000 (80.33)	224,212 (82.13)	2,668,434 (82.36)
Female	37,463 (13.99)	139,786 (14.78)	251,498 (19.51)	91,026 (19.60)	48,501 (17.77)	568,274 (17.54)
Missing data	386 (0.15)	753 (0.08)	1572 (0.12)	328 (0.07)	276 (0.10)	3315 (0.10)
Ethnicity/skin color^a^						
White	36,861 (13.77)	118,525 (12.53)	656,634 (50.94)	385,393 (83.00)	89,309 (32.72)	1,286,722 (39.71)
Black	12,864 (4.80)	56,634 (5.99)	116,338 (9.02)	19,221 (4.14)	15,127 (5.54)	220,184 (6.80)
Yellow	578 (0.22)	1423 (0.15)	6604 (0.51)	1332 (0.29)	780 (0.29)	10,717 (0.03)
Brown	205,475 (76.74)	685,338 (72.46)	457,305 (35.48)	48,223 (10.38)	156,288 (57.25)	1,552,629 (47.92)
Indigenous	4976 (1.86)	1484 (0.16)	777 (0.06)	1132 (0.24)	3151 (1.15)	11,520 (0.36)
Missing data	7003 (2.61)	82,446 (8.71)	51,415 (3.99)	9053 (1.95)	8334 (3.05)	158,251 (5.18)
Age group (years)						
0‐9	11,711 (4.37)	24,826 (2.62)	31,530 (2.45)	13,324 (2.87)	8856 (3.24)	90,247 (2.79)
10‐19	36,250 (13.54)	123,432 (13.05)	134,550 (10.44)	47,736 (10.28)	31,168 (11.42)	373,136 (11.52)
20‐39	134,872 (50.37)	459,366 (48.57)	528,599 (41.01)	187,859 (40.46)	122,273 (44.79)	1,432,969 (44.23)
40‐59	54,991 (20.54)	196,959 (20.82)	294,582 (22.85)	117,050 (25.21)	63,516 (23.27)	727,098 (22.44)
≥60	26,529 (9.91)	134,032 (14.17)	273,152 (21.19)	95,183 (20.50)	43,933 (16.09)	572,829 (17.68)
Missing data	3404 (1.27)	7235 (0.77)	26,660 (2.06)	3202 (0.68)	3243 (1.19)	43,744 (1.34)
Years of study						
<8 years	150,569 (56.23)	496,858 (52.53)	561,106 (43.53)	219,420 (47.25)	126,221 (46.24)	1,554,174 (47.97)
≥8 years	63,800 (23.83)	145,772 (15.41)	301,412 (23.38)	118,270 (25.47)	67,569 (24.75)	696,823 (21.51)
Missing data	53,388 (19.94)	303,220 (32.06)	426,555 (33.09)	126,664 (27.28)	79,199 (29.01)	989,026 (30.52)

a In the Brazilian context, the terms White (*branco*), Black (*preto*), Brown (*pardo*), Yellow (*amarelo*), and Indigenous (*indígena*) are the official racial/ethnic categories established by the Brazilian Institute of Geography and Statistics and are systematically used in national censuses and health information systems, including the Mortality Information System.

Table 2 shows that assaults (n=1,186,257, 36.61%) and transport accidents (n=860,386, 26.55%) were the main causes of death due to external factors in Brazil and its regions. Other significant causes were tumbles/falls (n=253,705, 7.83%), intentional self-inflicted injuries (n=240,843, 7.43%), and drowning (n=127,547, 3.94%). The North and Northeast regions recorded the highest rates of deaths from assaults, at 46% (n=123,167) and 43.8% (n=414,239), respectively. In contrast, transport accidents were the main cause in the South (n=151,712, 32.67%), and intentional self-inflicted injuries were the third leading cause in this region, exhibiting a higher percentage than other regions (n=57,138, 12.3%). In addition, 267,419 (8.25%) deaths were recorded as being of undetermined intent nationwide.

Regarding the time trend analyses, there was a slight decrease in standardized mortality rates in Brazil over the past 23 years, with an AAPC of −0.3 (95% CI −0.6 to −0.1; Table 3). Similarly, decreasing trends were observed in the Southeast (AAPC −1.8; 95% CI −1.9 to −1.6) and Central-West (AAPC −0.5; 95% CI −0.8 to −0.1) regions. In contrast, there was an increase in mortality rates in the Northeast (AAPC 1.2; 95% CI 0.9 to 1.6) and North (AAPC 0.8; 95% CI 0.5 to 1.2), while the South region showed stability (AAPC −0.2; 95% CI −0.4 to 0.1).

**Table 2.** Main causes of death from external causes in Brazil and its regions, 2000-2022.

Main causes of death from external causes	ICD-10^a^ codes	Frequency, n (%)
Brazil		
Assaults	X85-Y09	1,186,257 (36.61)
Transport accidents	V01-V99	860,386 (26.55)
Events with undetermined intent	Y10-Y34	267,419 (8.25)
Tumbles/falls	W00-W19	253,705 (7.83)
Self-inflicted intentional injuries	X60-X84	240,843 (7.43)
Accidental drowning and submersion	W65-W74	127,547 (3.94)
Other accidental risks to breathing	W75-W84	67,476 (2.08)
Accidental exposure to other specified and unspecified factors	X58-X59	58,475 (1.80)
Exposure to electric current, radiation, and extreme temperatures and pressures	W85-W99	33,144 (1.02)
Complications of medical and surgical care	Y40-Y84	32,011 (0.99)
Other causes	Other codes	112,760 (3.48)
North		
Assaults	X85-Y09	123,167 (46.00)
Transport accidents	V01-V99	70,789 (26.44)
Self-inflicted intentional injuries	X60-X84	16,566 (6.19)
Accidental drowning and submersion	W65-W74	16,210 (6.05)
Tumbles/falls	W00-W19	12,597 (4.70)
Events with undetermined intent	Y10-Y34	7,344 (2.74)
Exposure to inanimate mechanical forces	W20-W49	5,276 (1.97)
Exposure to electric current, radiation, and extreme temperatures and pressures	W85-W99	3,944 (1.47)
Other accidental risks to breathing	W75-W84	2,248 (0.84)
Accidental exposure to other specified and unspecified factors	X58-X59	1,915 (0.72)
Other causes	Other codes	7,701 (2.88)
Northeast		
Assaults	X85-Y09	414,239 (43.80)
Transport accidents	V01-V99	234,451 (24.79)
Events with undetermined intent	Y10-Y34	76,489 (8.09)
Self-inflicted intentional injuries	X60-X84	54,545 (5.77)
Tumbles/falls	W00-W19	50,182 (5.31)
Accidental drowning and submersion	W65-W74	40,984 (4.33)
Other accidental risks to breathing	W75-W84	15,218 (1.61)
Exposure to electric current, radiation, and extreme temperatures and pressures	W85-W99	13,322 (1.41)
Accidental exposure to other specified and unspecified factors	X58-X59	11,940 (1.26)
Complications of medical and surgical care	Y40-Y84	9,242 (0.98)
Other causes	Other codes	25,238 (2.67)
Southeast		
Assaults	X85-Y09	417,643 (32.40)
Transport accidents	V01-V99	314,813 (24.42)
Events with undetermined intent	Y10-Y34	155,007 (12.02)
Tumbles/falls	W00-W19	122,794 (9.53)
Self-inflicted intentional injuries	X60-X84	90,545 (7.02)
Accidental drowning and submersion	W65-W74	42,164 (3.27)
Accidental exposure to other specified and unspecified factors	X58-X59	37,820 (2.93)
Other accidental risks to breathing	W75-W84	36,401 (2.82)
Complications of medical and surgical care	Y40-Y84	17,661 (1.37)
Legal interventions and operations of war	Y35-Y36	11,555 (0.90)
Other causes	Other codes	42,670 (3.31)
South		
Transport accidents	V01-V99	151,712 (32.67)
Assaults	X85-Y09	131,577 (28.34)
Self-inflicted intentional injuries	X60-X84	57,138 (12.30)
Tumbles/falls	W00-W19	46,077 (9.92)
Events with undetermined intent	Y10-Y34	19,896 (4.28)
Accidental drowning and submersion	W65-W74	17,955 (3.87)
Other accidental risks to breathing	W75-W84	9,582 (2.06)
Exposure to inanimate mechanical forces	W20-W49	5,315 (1.14)
Exposure to smoke, fire, and flames	X00-X09	4,759 (1.02)
Exposure to electric current, radiation, and extreme temperatures and pressures	W85-W99	4,586 (0.99)
Other causes	Other codes	15,757 (3.39)
Central-West		
Assaults	X85-Y09	99,631 (36.50)
Transport accidents	V01-V99	88,621 (32.46)
Tumbles/falls	W00-W19	22,055 (8.08)
Self-inflicted intentional injuries	X60-X84	22,049 (8.08)
Accidental drowning and submersion	W65-W74	10,234 (3.75)
Events with undetermined intent	Y10-Y34	8,683 (3.18)
Other accidental risks to breathing	W75-W84	4,027 (1.48)
Exposure to electric current, radiation, and extreme temperatures and pressures	W85-W99	3,497 (1.28)
Exposure to inanimate mechanical forces	W20-W49	3,328 (1.22)
Accidental exposure to other specified and unspecified factors	X58-X59	2,411 (0.88)
Other causes	Other codes	8,453 (3.10)

a *ICD-10*: *International Classification of Diseases, 10th Revision*.

**Table 3.** Temporal trends of mortality rates from external causes by region, sex, and age group in Brazil, 2000-2022.

Variable and period		Segmented period, APC^b^ (95% CI)	Trend	Entire period, AAPC^c^ (95% CI)	Trend
Brazil				−0.3^a^ (−0.6 to −0.1)	Decreasing
2000‐2017		0.0 (−0.3 to 0.3)	Stable		
2017‐2020		−5.0 (−7.0 to 0.0)	Stable		
2020‐2022		5.0 (−1.5 to 8.9)	Stable		
Region					
North				0.8^a^ (0.5 to 1.2)	Increasing
2000‐2016		2.2^a^ (1.8 to 2.9)	Increasing		
2016‐2022		−2.8^a^ (−4.9 to −1.3)	Decreasing		
Northeast				1.2^a^ (0.9 to 1.6)	Increasing
2000 - 2014		2.9^a^ (2.3 to 3.7)	Increasing		
2014‐2022		–1.6^a^ (–3.3 to –0.4)	Decreasing		
Southeast				–1.8^a^ (–1.9 to –1.6)	Decreasing
2000-2003		–0.3 (–1.7 to 1.9)	Stable		
2003‐2007		–4.9^a^ (–6.5 to –3.7)	Decreasing		
2007‐2014		–0.9 (–1.4 to 0.7)	Stable		
2014‐2020		–3.3^a^ (–4.6 to –2.8)	Decreasing		
2020‐2022		4.6^a^ (1.8 to 6.5)	Increasing		
South				–0.2 (–0.4 to 0.1)	Stable
2000‐2004		2.4^a^ (0.8 to 6.0)	Increasing		
2004‐2017		–0.8^a^ (–1.2 to –0.4)	Decreasing		
2017‐2020		–4.6^a^ (–6.0 to –2.7)	Decreasing		
2020‐2022		6.0^a^ (2.4 to 9.2)	Increasing		
Central-West				–0.5^a^ (–0.8 to –0.1)	Decreasing
2000‐2004		2.0^a^ (0.4 to 6.7)	Increasing		
2004‐2007		–3.7^a^ (–5.5 to –0.9)	Decreasing		
2007‐2013		2.4^a^ (1.1 to 5.7)	Increasing		
2013‐2019		–4.1^a^ (–7.0 to –3.0)	Decreasing		
2019‐2022		0.9 (–1.8 to 4.9)	Stable		
Sex					
Males				–0.4^a^ (–0.7 to –0.1)	Decreasing
2000‐2017		0.0 (–0.3 to 0.3)	Stable		
2017‐2020		–5.2^a^ (–7.1 to -0.3)	Decreasing		
2020‐2022		4.6 (–1.6 to 8.3)	Stable		
Females				0.2 (0.0 to 0.5)	Stable
2000‐2007		–0.3 (–3.7 to 0.6)	Stable		
2007‐2012		1.8^a^ (0.8 to 3.3)	Increasing		
2012‐2020		–2.1^a^ (–2.8 to –1.6)	Decreasing		
2020‐2022		7.7^a^ (4.7 to 9.7)	Increasing		
Age group (years)					
0‐9				–1.5^a^ (–1.8 to –1.3)	Decreasing
2000‐2006		-4.3^a^ (-5.5 to -3.5)	Decreasing		
2006-2011		1.3^a^ (0.0 to 4.0)	Increasing		
2011‐2020		-3.5^a^ (–4.4 to –3.0)	Decreasing		
2020‐2022		9.9^a^ (5.3 to 12.9)	Increasing		
10-19				–1.1^a^ (–1.6 to –0.6)	Decreasing
2000‐2005		-2.0 (-8.2 to 0.7)	Stable		
2005‐2016		2.7^a^ (1.8 to 6.3)	Increasing		
2016‐2022		–6.9^a^ (–9.4 to –4.8)	Decreasing		
20-39				–0.4^a^ (–0.9 to –0.1)	Decreasing
2000‐2016		–0.1 (–2.3 to 0.6)	Stable		
2016‐2019		–5.2 (–7.6 to 2.6)	Stable		
2019‐2022		2.9 (–2.0 to 9.0)	Stable		
40-59				–0.5^a^ (–0.7 to –0.3)	Decreasing
2000‐2005		1.4^a^ (0.6 to 3.0)	Increasing		
2005‐2008		–4.7^a^ (–6.0 to –2.6)	Decreasing		
2008‐2012		1.6^a^ (0.4 to 3.7)	Increasing		
2012‐2019		–2.5^a^ (–4.1 to –1.9)	Decreasing		
2019‐2022		2.4^a^ (0.3 to 5.6)	Increasing		
≥60				1.2^a^ (0.8 to 1.8)	Increasing
2000‐2004		4.5^a^ (1.3 to 13.2)	Increasing		
2004‐2022		0.5 (–0.1 to 0.8)	Stable		

a *P*<.05 (statistically significant).

b APC: annual percentage change.

c AAPC: average annual percentage change.

In addition, a reduction in mortality rates among men (AAPC −0.4) and stability among women (AAPC 0.2) was verified. Most importantly, from 2020 onward, a significant increase in death rates among women was observed (AAPC 7.7). A decreasing trend in mortality rates across all age groups, except for the older adults (aged ≥60 years; AAPC 1.2), was also evident.

**Figure 2.**
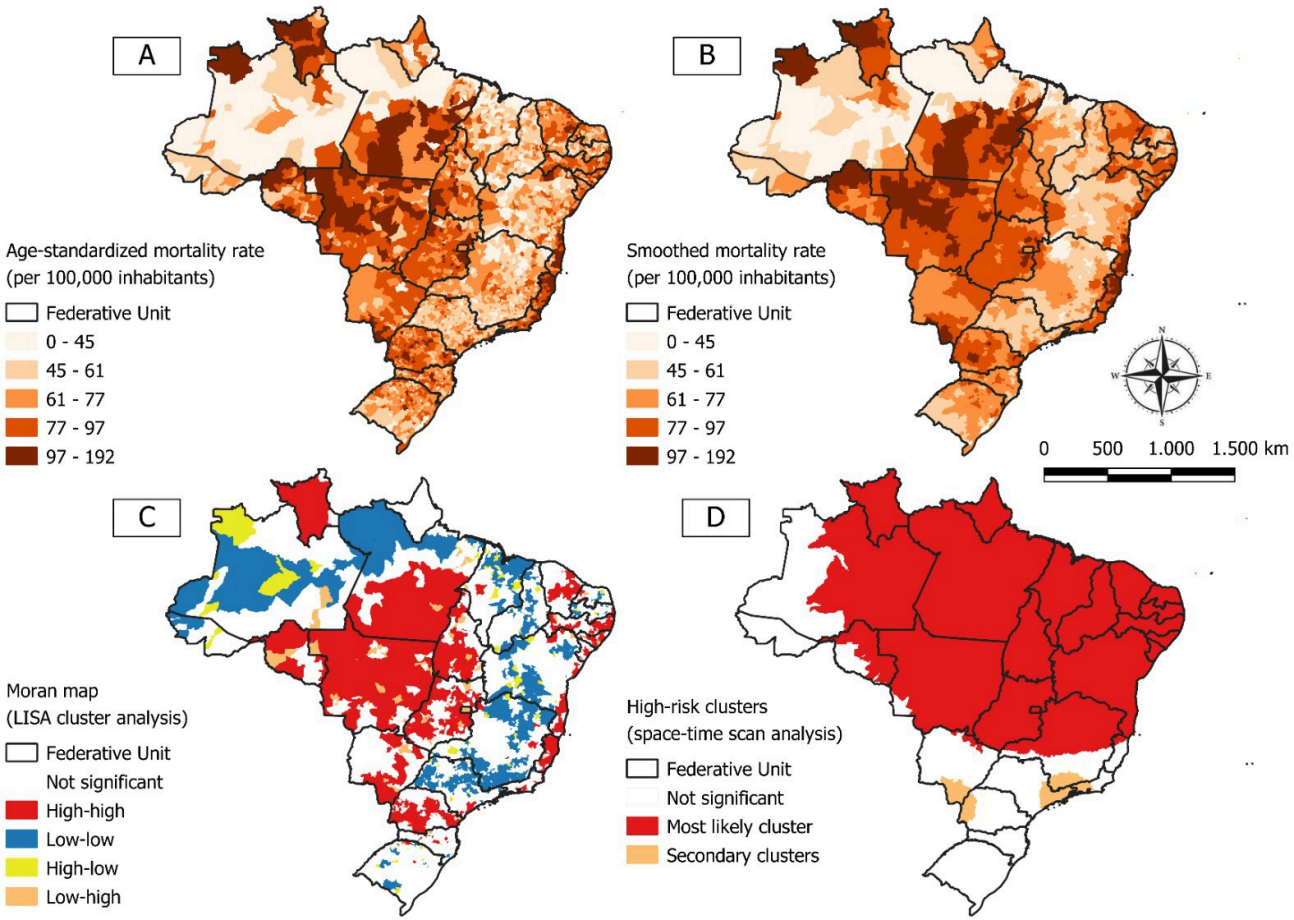
Spatial analysis of mortality rates from external causes in Brazil, 2000-2022. (A) Age-standardized mortality rate, (B) smoothed mortality rate, (C) Moran map (LISA cluster), and (D) spatiotemporal scanning analysis. LISA: Local Indicators of Spatial Association.

Figure 2A–B shows maps of the spatial distribution of standardized and smoothed mortality rates due to external causes in Brazilian municipalities. Considering standardized rates, areas with high mortality (>97/100,000 inhabitants) were detected across all regions of the country. Nevertheless, when assessing the smoothed rates, municipalities with high mortality were mainly concentrated in the North and Central-West regions. A positive and significant spatial autocorrelation of mortality from external causes in Brazil was found (Moran I 0.53; *P*=.001). These results confirm the existence of spatial dependence in mortality from accidents and violence among Brazilian municipalities. In addition, 893 municipalities with high mortality rates (high and high–in red), forming clusters with a high risk of death due to violent and accidental incidents—especially in the North, Central-West, and Northeast regions—were identified (Figure 2C).

Finally, the spatiotemporal scanning analysis identified the formation of 3 statistically significant clusters of high-risk mortality from external causes. Notably, the primary cluster presented a relative risk of 1.22 and included a large portion of the national territory, encompassing 2922 municipalities and 21 out of 27 federative units, mainly located in the North, Northeast, and Central-West regions (Figure 2D and Table 4).

**Table 4.** Spatiotemporal scan analysis of mortality rates from external causes in Brazil, 2000-2022.

Cluster	Time period	Municipalities, n	States^a^	Observed	Expected	RR^b^	LLR^c^	*P* value
1	2012‐2022	2922	AM, AP, RR, RO, PA, TO, MA, PI, CE, RN, PB, PE, AL, SE, BA, MG, ES, GO, DF, MT, MS	844,818	725,563	1.22	12,233.58	.001
2	2000‐2003	299	MG, RJ, SP	147,643	113,725	1.31	4806.37	.001
3	2003‐2013	81	MS, PR	21,915	15,341	1.43	1248.35	.001

a AM: Amazonas; AP: Amapá; RR: Roraima; RO: Rondônia; PA: Pará; TO: Tocantins; MA: Maranhão; PI: Piauí; CE: Ceará; RN: Rio Grande do Norte; PB: Paraíba; PE: Pernambuco; AL: Alagoas; SE: Sergipe; BA: Bahia; MG: Minas Gerais; ES: Espírito Santo; GO: Goiás; DF: Distrito Federal; MT: Mato Grosso; MS: Mato Grosso do Sul; RJ: Rio de Janeiro; SP: São Paulo; PR: Paraná.

b RR: relative risk.

c LLR: log likelihood ratio.

## Discussion

### Principal Findings

To the best of our knowledge, this is the first study to assess the dynamics of mortality from external causes across all regions of Brazil, applying different statistical and spatiotemporal techniques over an extensive period of 23 years. The findings indicate a slight decrease in the mortality rate from violent and accidental incidents in Brazil during this period. Furthermore, a reduction or stability was identified in almost all regions and population groups, except for the North and Northeast regions and the older adults, where increasing trends in these deaths were observed. Taken together, these results highlight the concerning scenario of deaths from external causes in Brazil and the challenges the country will face in achieving the goals of the DANT Plan, agreed upon with the WHO by 2030 [2].

Notably, accidents and violence are considered serious public health problems and are among the leading causes of physical disability and mortality worldwide [1]. Faced with the dramatic magnitude of this phenomenon, several countries have implemented measures to reduce the number of incidents and deaths from these causes, such as the development of public safety policies and programs, the enactment of stricter laws [20,21], increased traffic inspection and education, and improvements in road infrastructure [22].

In this context, Brazil has also implemented strategies to mitigate these problems and reduce the number of deaths from external causes. In addition to the Dant Plan, other initiatives have been adopted, such as the National Policy for Reducing Morbidity and Mortality from Accidents and Violence [23], the Disarmament Statute [24], the implementation of qualified public safety actions and programs by some municipalities and states [25], and the National Policy for the Prevention of Self-Mutilation and Suicide [26]. Safety awareness campaigns have also been implemented, and more severe penalties have been applied for traffic violations across the country [27-29].

As also shown in previous studies [25,30,31], we strongly believe that these measures may have contributed to the reduction of mortality rates from violence and accidents in Brazil, as identified in this study. Nonetheless, it is important to highlight that significant and lasting changes in these rates occur slowly and gradually, and the general positive effects are intrinsically related to the consistency and effectiveness of these interventions. These effects can only be perceived in the long term [8].

Remarkably, reductions in death rates from external causes do not occur equally across different regions of the world. The problem involving external causes is complex and can be influenced by a variety of socioeconomic, demographic, and political factors [1]. Similarly, in Brazil, regions with greater socioeconomic vulnerability, such as the North and Northeast, showed an increase in mortality due to accidents and violence. Brazil has continental dimensions and is one of the most unequal countries in the world [11]. As a result, these disparities can influence rates and patterns of deaths from external causes across regions [1]. Unfortunately, certain population groups and regions are more affected, and the highest death rates are observed especially in low-income areas [32] and in those where prevention policies and strategies are absent or ineffective [33].

Regarding sociodemographic factors, a significant percentage of deaths occurred among men and young adults (aged 20-39 years). These findings are consistent with previous studies that identified men and young adults as the groups most affected by violent and accidental incidents [34,35]. Men and young adults are more vulnerable to deaths from external causes, largely due to their lifestyles, which often expose them to individual risk factors for accidents and violence, such as greater involvement with alcohol, drugs, firearms, participation in organized crime [9,36], and disregard for traffic laws and regulations [35]. This scenario has a significant impact on society, leading to the loss of economic and intellectual potential, which can hinder economic growth and delay the development of a region or country [2].

On the other hand, mortality from external causes among women showed a significant increase from 2020 onward. This increase is likely related to the evolution of women’s roles in Brazilian society, leading them to occupy the same spaces and adopt lifestyles similar to those of men, which can increase risk behaviors for accidents and violence [37]. Furthermore, some authors believe that the reduction in the federal public budget for policies to combat violence against women, along with the encouragement of radical and conservative politicians, helped strengthen patriarchy and made access to firearms more flexible from 2019 to 2022, which likely contributed to the intensification of gender-based violence against women and femicide in recent years [25,38].

There was also an increasing trend in mortality rates from external causes among older adults, which can be attributed, in part, to the increase in life expectancy and the growth of this population in Brazil during recent decades. Older adults, due to the processes of senescence and senility, may experience a decrease in physical and cognitive capabilities, making them more susceptible to incidents such as falls [39], drownings [40], and traffic accidents [41]. Furthermore, aging can increase the occurrence of health conditions and stressors that raise the risk of suicide, such as a higher prevalence of mental disorders, marital problems, family losses, and social isolation [42].

As expected, assaults and transport accidents accounted for 63.16% of all deaths from external causes in Brazil and its regions. Undeniably, Brazil is widely recognized as one of the most violent countries in South America, being part of the group of nations with the highest risk of homicides [43]. This alarming scenario of violence is related to a series of factors, with social inequities emerging as a key element. Homicide rates are closely linked to structural inequalities, and violence is particularly intense in poorer areas, neglected urban spaces, or peripheral regions, such as favelas, where the population faces greater exposure to factors such as involvement with drugs, weapons, trafficking, and organized crime [9,44].

As for traffic accidents, several factors contribute to the high death rates in Brazil. In recent years, the country has seen exponential growth in the fleet of motor vehicles and motorcycles [45]. However, this increase has not been accompanied by improvements in road infrastructure, and many highways remain poorly maintained, with inadequate signage and a lack of safety devices, which considerably increases the risk of accidents [46]. Even more concerning, risky behaviors are quite common among drivers in the country, including driving under the influence of alcohol and drugs, speeding, and neglecting the proper use of safety equipment such as helmets and seat belts [47].

Spatial analysis and spatiotemporal scanning techniques used in this study enabled the identification of areas with a high risk of death related to external causes, concentrated mainly in the North, Northeast, and Central-West regions of Brazil. Previous studies have already classified these areas as having a high concentration of deaths from external causes [7,48].

In fact, the North, Northeast, and Central-West regions exhibited high-risk areas for mortality from external causes, and several factors are intrinsically linked to this concerning scenario. These Brazilian regions have experienced a process of rural exodus and intense, disorganized urbanization, which has driven the emergence of deprived areas lacking adequate public policies and infrastructure. As a result, there has been an increase in social inequities and structural determinants related to interpersonal and self-inflicted violence [49,50]. In addition, the migration of criminal factions from the Southeast region to states in the North and Northeast, along with the presence of border areas in the Central-West dominated by drug traffickers, has contributed to the expansion of the drug trafficking market, areas of armed conflict, and, ultimately, increased the risk of violent deaths [51]. Some states in the North and Central-West also face high rates of rural conflicts and territorial disputes, especially between Indigenous communities and large cattle ranchers and farmers [52,53]. Unfortunately, these conflicts result in an alarming number of violent deaths each year, especially among Indigenous people and rural producers, and significantly contribute to the increase in deaths from external causes in these regions of Brazil.

Despite advances in recent years, mortality from external causes remains a major public health challenge in Brazil. Accidents and violence are preventable, and it is the responsibility of policymakers and government officials to take effective measures to reduce them [8]. In this context, it is essential to improve current legislation and public security policies to combat drug trafficking and organized crime, as well as to enforce stricter regulation of civilian gun ownership [54,55]. It is equally important to strengthen violence prevention programs, including those targeting vulnerable populations such as women, Black people, and Indigenous communities [25], alongside expanding traffic enforcement and improving road infrastructure [22]. Other essential strategies may include improving urban mobility to reduce the risk of falls among older adults [56], ensuring adequate access to mental health services for individuals in psychological distress, and strengthening preventive actions against suicide [26].

### Implications for Public Health and Surveillance

This study provides critical insights into the persistent public health challenge posed by mortality from external causes across Brazil. The findings emphasize the importance of targeted, region-specific public health interventions, particularly in high-risk areas such as the North, Northeast, and Central-West regions, where socioeconomic disparities and insufficient infrastructure significantly contribute to mortality rates.

The spatiotemporal patterns observed suggest the need for continuous and enhanced public health surveillance systems capable of detecting changes in mortality trends and risk factors specific to different demographic groups, such as young adults, men, and, increasingly, older adults. Surveillance systems that incorporate real-time data, predictive analytics, and spatial analysis will be essential in enabling timely, evidence-based public health responses to prevent violence and accidents.

Public policy makers and health program developers should consider these findings when planning interventions that address the intersection of multiple social determinants of health influencing mortality patterns, by adopting strategies that integrate cartographic approaches and analyses. In addition, efforts to prevent external causes of death should prioritize stricter enforcement of traffic laws, the development of violence prevention programs, and the implementation of policies targeted at high-risk populations and geographic areas. Enhanced public health strategies focused on injury and violence prevention, coupled with robust surveillance systems, are fundamental for Brazil to achieve its health targets under the WHO’s DANT plan by 2030, improving overall population health and reducing preventable mortality across the country.

### Limitations

This study has some limitations that should be acknowledged. Because secondary data were used, their quality cannot be guaranteed, and the values used in the analyses could be either underestimated or overestimated. As this is an ecological study, the results observed at the group level may not precisely reflect what occurs at the individual level. However, despite these limitations, the results described herein still provide valuable insights into the spatiotemporal panorama of mortality from external causes in Brazil and can support the development of public policies and interventions aimed at reducing mortality, particularly in high-risk areas.

### Conclusion

Taken together, the results showed a slight reduction in mortality from external causes in Brazil. However, there was an increase in death rates in the North and Northeast regions of the country and among older adults, alongside high mortality rates among men and young adults. In addition, assaults and transport accidents remained the leading causes of death across all regions. Spatial analysis and spatiotemporal scanning revealed that the distribution of deaths from these causes is heterogeneous across Brazilian territory, with the primary risk areas concentrated in the North, Northeast, and Central-West regions. Given this scenario, we emphasize the need for intersectoral public policies that cover the entire national territory. These policies must involve greater allocation of resources and strategic targeting of prevention and control measures, focusing efforts on the most critical areas. Only in this way can the country achieve the goals of the Dant Plan, agreed with the WHO by 2030.

## Abbreviations

AAPC: average annual percentage change
APC: annual percentage change
DC: death certificate
*ICD-10*: *International Statistical Classification of Diseases and Related Health Problems, Tenth Revision*
SIM: Mortality Information System
WHO: World Health Organization
